# Supplementary figures and images for: Transcriptome profiling of human col\onic cells exposed to the gut pathobiont Streptococcus gallolyticus subsp. gallolyticus
Source: PLoS One. 2023 Nov 30;18(11):e0294868. doi: 10.1371/journal.pone.0294868 (PMC10688619; doi:10.1371/journal.pone.0294868)

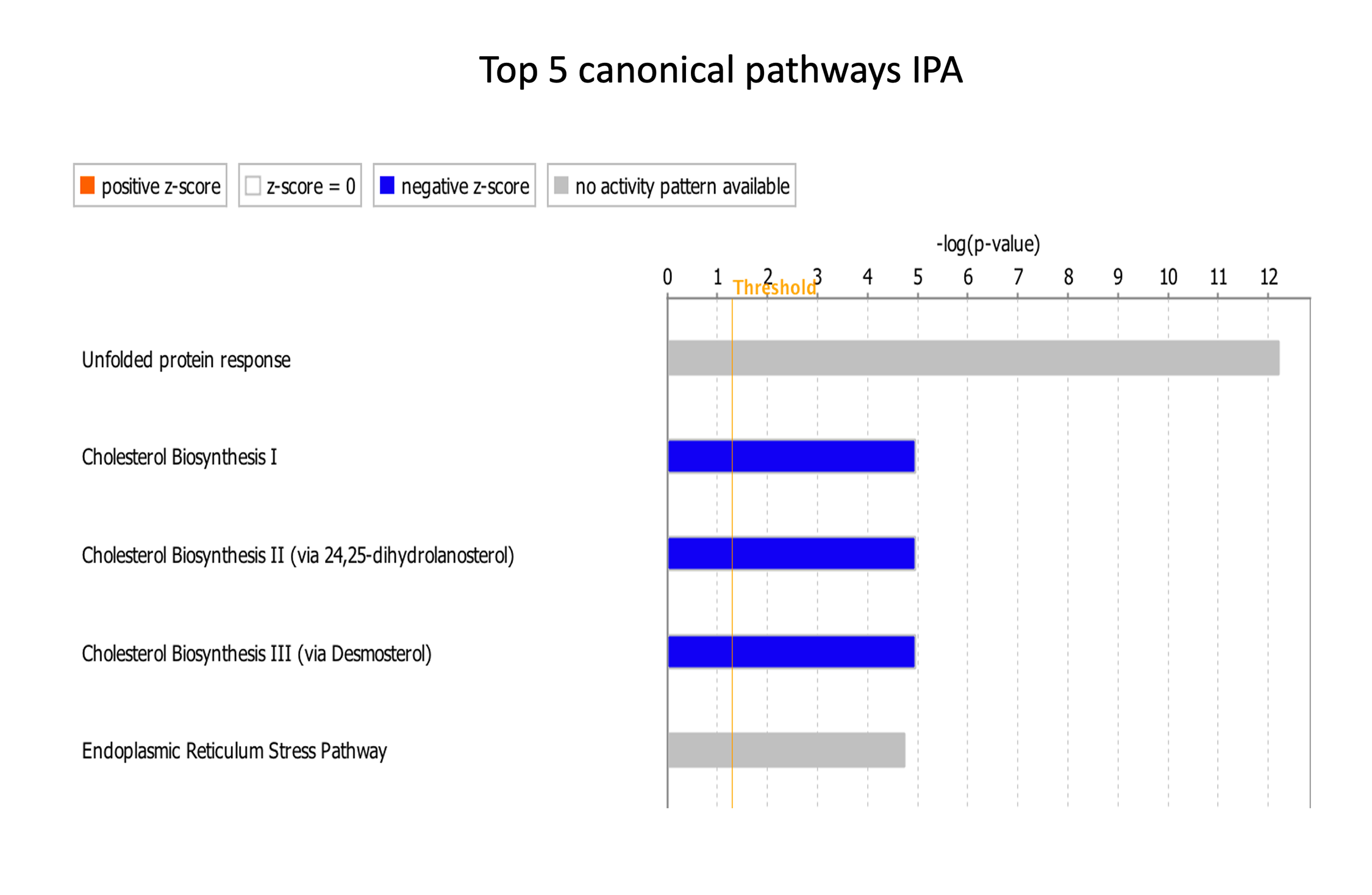

Supplement: S1 Fig — The data set used in this analysis was genes differentially expressed between SGG UCN34 and SGM detected in HT29 after 24 h of infection. Canonical pathways that were most significant to the data set were identified from the QIAGEN Ingenuity Pathway Analysis library of canonical pathways. Canonical pathways with p-values < 0.05 (Fischer’s exact test) were statistically significant. The activation Z-score was calculated to predict activation or inhibition of transcriptional regulators based on published findings accessible through the Ingenuity knowledge base. Regulators with Z-score greater than 2 (positive Z-score) or less than −2 (negative Z-score) were significantly activated (orange) or inhibited (blue). Regulators with Z-score of 0 are represented in white and those for which the Z-score couldn’t be calculated are shown in grey. (TIF) [file pone.0294868.s001.tif]

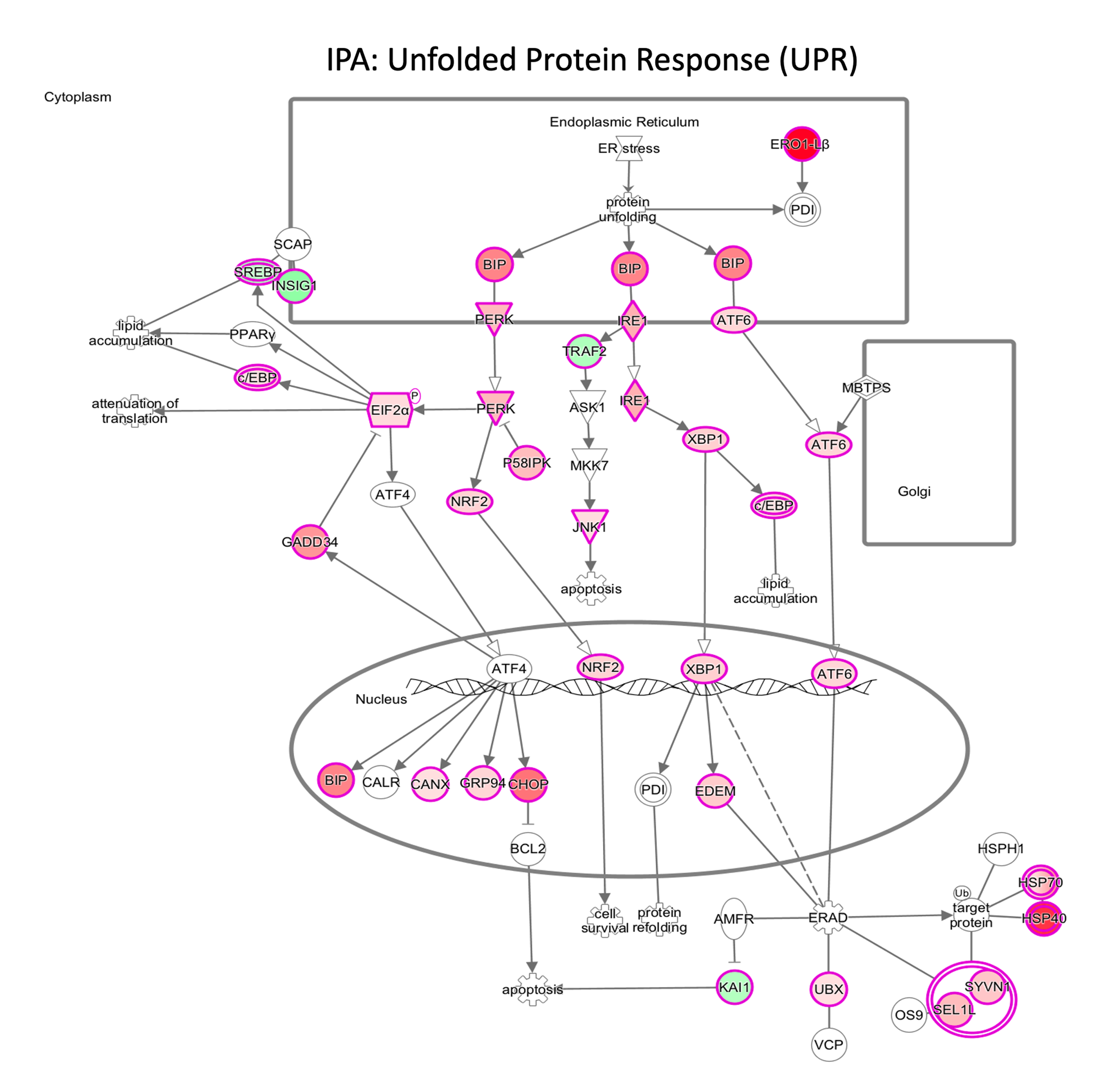

Supplement: S2 Fig — Differentially expressed genes in HT29 cells after 24 h of infection with SGG UCN34 vs SGM. Nodes represent molecules in a pathway, while the biological relationship between nodes is represented by a line (edge). Edges are supported by at least one reference in the Ingenuity Knowledge Base. The intensity of color in a node indicates the degree of up- (red) or down- (green) regulation. Nodes that are red and green represent the increased and decreased measurements respectively. Nodes in orange represents predicted (hypothetical) activation and nodes in blue predicted (hypothetical) inhibition. Nodes are displayed using shapes that represent the functional class of a gene product (Circle = Other, Nested Circle = Group or Complex, Rhombus = Peptidase, Square = Cytokine, Triangle = Kinase, Vertical ellipse = Transmembrane receptor). Edges are marked with symbols to represent the relationship between nodes (Line only = Binding only, Flat line = inhibits, Solid arrow = Acts on, Solid arrow with flat line = inhibits and acts on, Open circle = leads to, Open arrow = translocates to). An orange line indicates predicted upregulation, whereas a blue line indicates predicted downregulation. A yellow line indicates expression being contradictory to the prediction. Gray line indicates that direction of change is not predicted. Solid or broken edges indicate direct or indirect relationships, respectively. (TIF) [file pone.0294868.s002.tif]

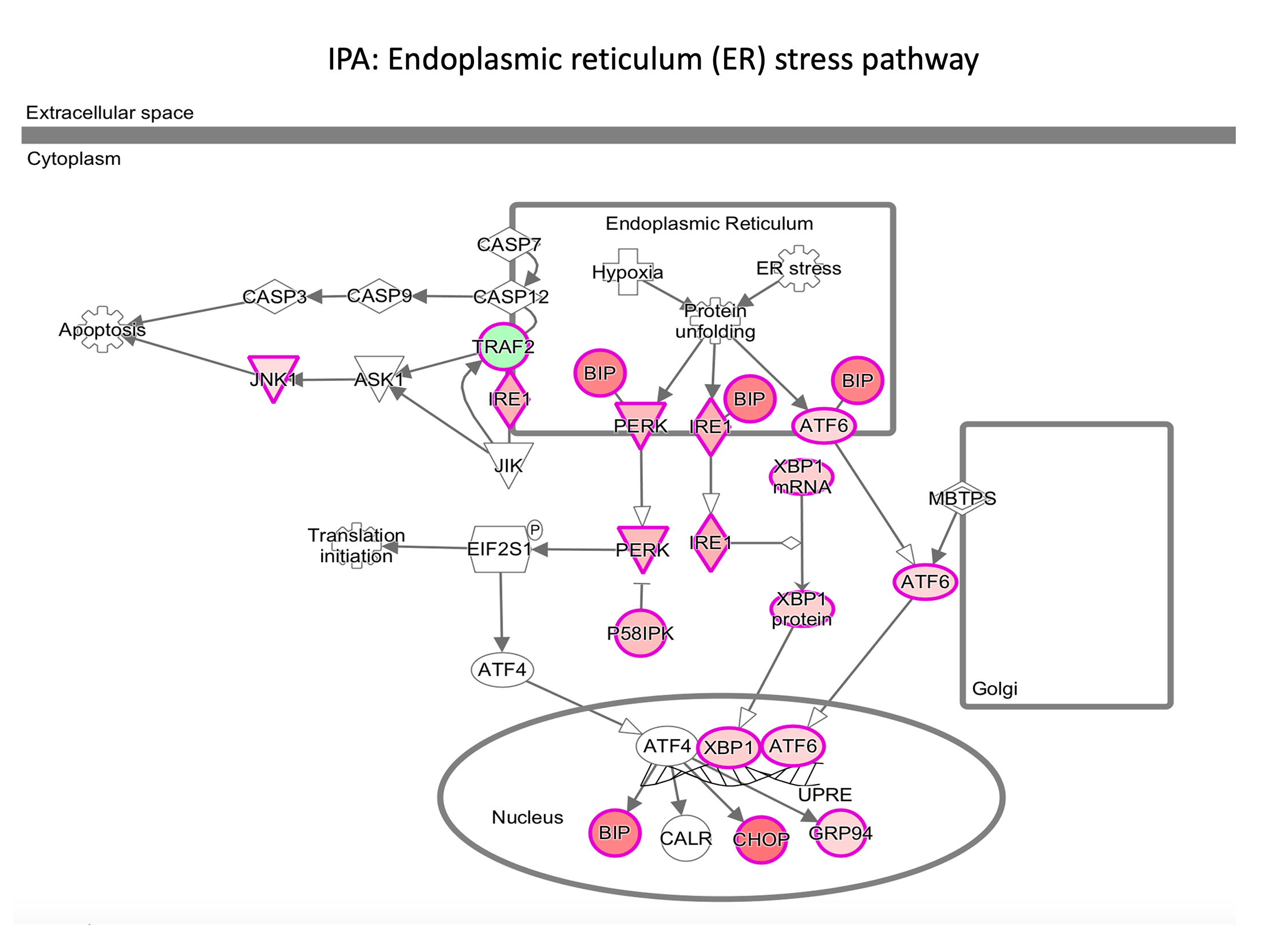

Supplement: S3 Fig — The data set used in this analysis was differentially expressed genes in HT29 cells after 24 h of infection with SGG UCN34 vs SGM. Nodes represent molecules in a pathway, while the biological relationship between nodes is represented by a line (edge). Edges are supported by at least one reference in the Ingenuity Knowledge Base. The intensity of color in a node indicates the degree of up- (red) or down- (green) regulation. Nodes that are red and green represent the increased and decreased measurements respectively. Nodes in orange represents predicted (hypothetical) activation and nodes in blue predicted (hypothetical) inhibition. Nodes are displayed using shapes that represent the functional class of a gene product (Circle = Other, Nested Circle = Group or Complex, Rhombus = Peptidase, Square = Cytokine, Triangle = Kinase, Vertical ellipse = Transmembrane receptor). Edges are marked with symbols to represent the relationship between nodes (Line only = Binding only, Flat line = inhibits, Solid arrow = Acts on, Solid arrow with flat line = inhibits and acts on, Open circle = leads to, Open arrow = translocates to). An orange line indicates predicted upregulation, whereas a blue line indicates predicted downregulation. A yellow line indicates expression being contradictory to the prediction. Gray line indicates that direction of change is not predicted. Solid or broken edges indicate direct or indirect relationships, respectively. (TIF) [file pone.0294868.s003.tif]
